# Influence of Molecular Weight on the Enzymatic Degradation of PLA Isomer Blends by a Langmuir System

**DOI:** 10.3390/ma16145087

**Published:** 2023-07-19

**Authors:** Donghyeok Im, Vishal Gavande, Hak Yong Lee, Won-Ki Lee

**Affiliations:** 1 Department of Polymer Engineering, Pukyong National University, Busan 48513, Republic of Korea; dhlim@kiflt.re.kr (D.I.); vgawande77@gmail.com (V.G.); 2 Fine & Specialty Chemical Research Group, Korea Institute of Footwear & Leather Technology, Busan 47154, Republic of Korea; 3 Research Center for Bio-Based Chemistry, Korea Research Institute of Chemical Technology, Ulsan 44429, Republic of Korea; lee.104hy@gmail.com

**Keywords:** biodegradable polymer, stereochemical polylactides, stereocomplex, enzymatic degradation, Langmuir monolayer

## Abstract

Polylactides (PLAs) and lactide copolymers are biodegradable, compostable, and derived from renewable resources, offering a sustainable alternative to petroleum-based synthetic polymers owing to their advantages of comparable mechanical properties with commodity plastics and biodegradability. Their hydrolytic stability and thermal properties can affect their potential for long-lasting applications. However, stereocomplex crystallization is a robust method between isomer PLAs that allows significant amelioration in copolymer properties, such as thermal stability, mechanical properties, and biocompatibility, through substantial intermolecular interactions amid l-lactyl and d-lactyl sequences, which have been the key approach to initial degradation rate and further PLA applications. It was demonstrated that the essential parameters affecting stereocomplexation are the mixing ratio and the chain length of each unit sequence. This study deals with the molecular weight, one of the specific interactions between isomers of PLAs. A solution polymerization method was applied to control molecular weight and chain architecture. The stereocomplexation was monitored with DSC. It was confirmed that the lower molecular weight polymer showed a higher degradation rate, as a hydrolyzed fragment having a molecular weight below a certain length dissolves into the water. To systematically explore the critical contribution of molecular weights, the Langmuir system was used to observe the stereocomplexation effect and the overall degradation rate.

## 1. Introduction

For several decades, conventional petroleum-derived synthetic polymers have been favored materials extensively employed in various industrial and consumer applications. This preference is primarily due to their remarkable mechanical strength, toughness, long-term stability, and cost-effectiveness [1,2]. However, the accumulation of non-degradable plastic waste poses a significant threat to the environment. The global production of synthetic polymers, reaching approximately 140 million tons annually, has made them an integral part of our daily lives [3,4]. The extensive utilization of petrochemical-based synthetic polymers for over a century has resulted in significant environmental concerns, including the accumulation of non-degradable waste in landfills and oceans. Consequently, substantial investments are being made worldwide to either improve the disposal methods of petrochemical-based polymers or seek alternative materials that are more environmentally friendly. In response to this crucial challenge, significant efforts have been dedicated to the development of sustainable polymers, aiming for a more ecologically conscious future [5,6,7,8,9].

Promising biodegradable polymers such as polylactide (PLA), poly(butylene succinate) (PBS), poly(3-hydroxybutyrate), and poly(butylene adipate-*co*-terephthalate) (PBAT) have been identified among various options. These polymers are capable of undergoing degradation through ester bond cleavage under acidic, basic, or enzymatic conditions [2,10,11,12,13]. PLA, in particular, holds a prominent position in the market with its large-scale industrial production. However, certain challenges still hinder their widespread utilization, including issues of brittleness, as well as limited thermal and hydrolytic stability. Various physical and chemical treatments have been applied, such as blending, stereocomplexation, and adding nanomaterials, to overcome these properties [14,15,16]. The most conventional and robust way to prepare PLA with significant properties is via stereocomplex crystallization using enantiomeric pairs of poly(l-lactide) (PLLA) and poly(d-lactide) (PDLA), which can prevail from both melt and solution blending [17]. The blend of enantiomeric PLLA and PDLA provides strengthened mechanical and thermal properties due to strong intersegment interactions, resulting in more tightly packed chain confirmations within the stereocomplex racemic crystal lattice [18], and thus improving mechanical properties, thermal stability, and hydrolytic resistance compared to its parent enantiomeric pure [19,20].

**Figure 1:**
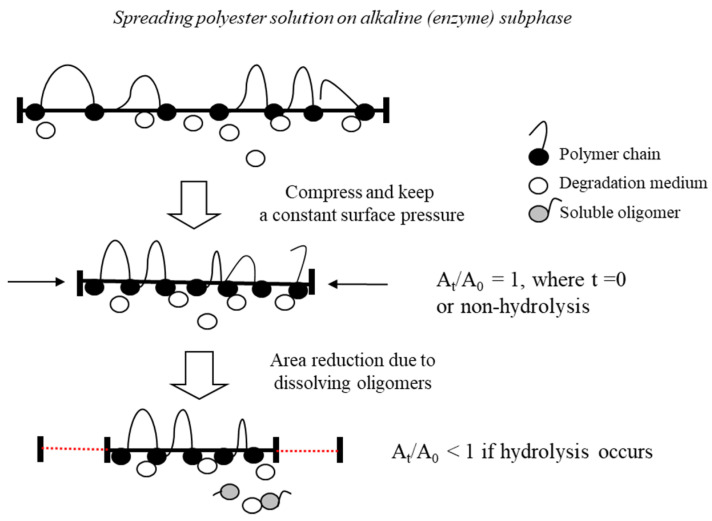
Schematic diagram of the degradation kinetics of the Langmuir monolayer.

The degradability of biodegradable polymers is one of their most significant characteristics because of the lifespan of marketed products. Fredericks et al. reported that the 1.7% weight loss of biodegradable polyester decreases 66% of its mechanical properties [21]. Therefore, the mechanical properties of biopolyesters are adversely affected during the initial degradation. Many researchers have reported that the initial degradation of biopolyesters is regulated by the physical accessibility of the polymer structure to abiotic conflicts [2,9,11,14,21]. Thus, it is essential to control the rate of degradation when designing commercially graded biomaterials. The initial degradation rate can be adjusted by chemical or physical modifications such as blending, plasma processing, and specific interactions. The Langmuir–Blodgett technique is a method of making monolayers with a controlled layered structure and is conceivably the best method to manipulate materials at the molecular level. The fundamental advantage of the Langmuir technique is that it can control the lateral packing density in monolayers, which is not feasible with any other conventional method [22,23]. This technique involves a quantitative investigation of the in situ enzymatic or hydrolyzable degradation rate of polyester monolayers (Figure 1).

In this study, we have studied the effect of molecular weight on stereocomplexation. A solution polymerization method was introduced for synthesizing the desired molecular weights of PLLA and PDLA. The different molecular weights of PLA were synthesized according to the polymerization time, an appropriate amount of catalyst, and solvent. The stereocomplexation was validated by DSC, and the degradation rate of the stereocomplex systems was determined at the molecular level through the Langmuir technique.

## 2. Experimental

### 2.1. Materials

l- and d-lactide were provided by Purac, based in Gorinchem, the Netherlands. Tin(II) 2-ethylhexanoate, proteinase K, and Tris(hydroxymethyl)amino-methane were obtained from Sigma-Aldrich, located in St. Louis, MO, USA. Solvents such as toluene, chloroform, methanol, *n*-hexane, and hydrochloric acid were purchased from Samchun, a supplier in Pyungtaek, Republic of Korea. All chemicals were utilized as received without additional purification.

### 2.2. Synthesis of PLA

Enantiomeric PLAs were synthesized by solution polymerization at 70~120 °C in toluene for 12~48 h using tin(II) 2-ethylhexanoate (0~10 wt% of lactide) as a catalyst in a nitrogen atmosphere. This method allows synthesizing PLA directly from the lactide monomer and can produce a desired molecular weight by varying the amount of catalyst, polymerization temperature, and reaction time [24]. This procedure accounts for a high-boiling-point solvent, which helps to remove water as a byproduct. To obtain a high-molecular-weight polymer, it is imperative to withdraw water at a high temperature during the polymerization.

The polymerization of LA was performed in toluene, and the initial concentration of the monomer was 30 wt%. A high concentration of the catalyst is typically used to initiate the faster reaction kinetics, leading to shorter reaction times and higher polymerization yields. It is difficult to control the molecular weight for a lower catalyst concentration (<1 wt%) as the number of reaction sites decreases dramatically, making it difficult to proceed with the reaction. Lower catalyst concentrations can slow down the reaction rate, resulting in longer polymerization times. This can be advantageous when aiming for specific molecular weights or controlling the polymerization process more precisely. By reducing the catalyst concentration, the reaction can proceed at a slower pace, allowing for better control over molecular weight, polymerization extent, and final PLA properties. The ring-opening polymerization in solvent was conducted at 100 °C for several hours under an inert atmosphere because the polymerization at a low reaction temperature below 80 °C did not occur. However, the evaporation of the solvent occurs too quickly, with or without reflux, when the reaction temperature surpasses 110 °C, which makes polymerization difficult. It indicated that solution polymerization is an easier process than bulk polymerization for controlling the molecular weight of PLA. Although many factors are accounted for to perform the polymerization, they require various trials and errors. The polydispersity index (PDI) of PLAs synthesized by solution polymerization was much narrower than that of bulk polymerization due to the minimization of chain transfers. A lower PDI indicates a more narrow and homogeneous distribution of molecular weights in the synthesized polymer. A PDI below 2 suggests that the majority of polymer chains have similar lengths, resulting in a more uniform material. This can be advantageous in various applications, as it indicates a higher level of control over the polymerization process and a more consistent product.

After the solution polymerization, a rotary evaporator was used to remove the excess solvent. The obtained polymers were purified by the dissolution/precipitation method using chloroform as a solvent and methanol and *n*-hexane as non-solvents. Subsequently, the products were kept for drying at 25 °C for about three days. For studying enzymatic degradation, a spin coating process was used for making thin films with 5 wt% polymer solution, and 1 μmol/mL polymer solution was spread on a subphase for the Langmuir film.

### 2.3. Measurements

The gel permeation chromatography (GPC) analysis was conducted to determine the average molecular weight (M_n_) and PDI (M_w_/M_n_). The GPC system used was the CBM-20A, DGU-20A3, LC-6AD, SIL-20ACHT, CTO-20A, RID-10A by Shimadzu Corp. The analysis was performed in chloroform at a flow rate of 1.0 mL/min at a temperature of 40 °C. Polystyrenes from Showa Denko K.K. (Shodex^®^ STANDARD SM-105) were employed as standard references. The thermal properties of the samples were assessed using a differential scanning calorimeter (DSC 1, Mettler Toledo) under a nitrogen atmosphere, with a heating rate of 10 °C/min. Table 1 contains a list of the synthesized polymers along with their respective reaction parameters and physical properties utilized in this study. For the experiments, deionized water (DI) with a resistivity of 18.2 MΩ·cm was obtained from a UNIONS ultrapure water system, provided by Sinhan Science. Prior to use, the Langmuir trough (580 × 145 × 4 mm^3^, KSV-NIMA Langmuir probe system, Biolin Scientific) was stabilized for one hour and filled with approximately 500 mL of deionized water. The solution, typically 100 μL of 1 μmol/mL concentration, was spread on the water surface for surface pressure-area (π-A) isotherm and kinetic measurements, unless otherwise specified. The solvent was allowed to evaporate for 1 min, as determined based on the reproducibility of the π-A curve. The compression rate of the barriers was maintained at 10 cm^2^/min for all experiments. Scanning electron microscopy (SEM) was employed to obtain surface morphological images using the Tescan-MIRA3 microscope from the Czech Republic.

### 2.4. Enzymatic Degradation

The buffer solution was prepared with deionized water and 0.1 M Tris(hydroxymethyl) aminomethane. The pH of the solution was adjusted to 8.6 with the addition of hydrochloric acid. Enzymatic degradation experiments were conducted using proteinase K, with 0.02 mg and 0.1 mg enzyme quantities in the buffer solution at room temperature for monolayer and thin film experiments, respectively. PLLA and PDLA homopolymer solutions in chloroform were prepared by vigorously stirring for 24 h at a concentration of 1 μmol/mL, and each solution was mixed. The Langmuir trough was stabilized for 1 h and then filled with approximately 500 mL of deionized water. After spreading the polymer solution, a waiting time of over 1 min was employed, determined based on the reproducibility of the π-A curve, allowing the solvent to evaporate.

For the investigation of enzymatic degradation on spin-coated samples, a cuvette containing 1 mL of buffer solution with an enzyme concentration of 0.1 mg/mL was used. After degradation, the samples were washed with distilled water and dried at 25 °C for 24 h.

### 2.5. Stereocomplex Effect

As mentioned above, the synergistic effect on the mechanical and thermal properties can be achieved by the stereocomplexation between the isomers of PLA. There are some factors that influence the stereocomplexation, such as molecular weight, blending conditions and ratios, and optical purity. However, achieving a perfect stereocomplexation without any homocrystallization of PLLA or PDLA is not possible in all blends with low, high, or medium molecular weights. The molecular weights of PLLA and PDLA play a significant role in determining the competition between the formation of stereocomplexes and homocrystallites in their enantiomeric blends [25]. There is a critical molecular weight of approximately 10^5^ g/mol, beyond which the formation of stereocomplexes becomes challenging [18]. Since both enantiomers of PLLA and PDLA need to be combined in stereocomplexation, they require a larger diffusion path compared to regular homocrystallization. To systematically investigate the critical contributions of molecular weights, crystallization temperature, chain length, and molar ratios to the formation of stereocomplexes, our research group conducted a quantitative preparation of a wide range of PLA racemic blends, encompassing various molecular weights of the enantiomers.

**Figure 2:**
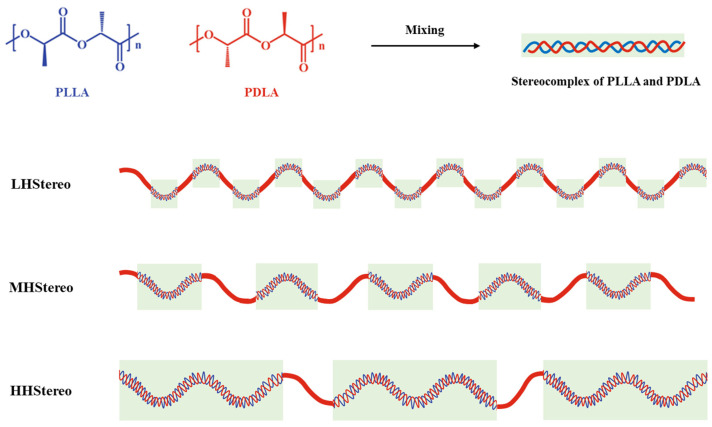
Schematic diagram of stereocomplexation according to molecular weight.

Additionally, the hydrolytic degradation and enzymatic degradation (from proteinase K) of stereocomplex PLA are more delayed than PLA. In this study, the effect of the molecular weight of each component on stereocomplex formation was systematically investigated. Four (PLLA/PDLA) blend systems, LL, LH, MH, and HH, were selected as shown in Figure 2, where the blend code LH indicates a low-molecular-weight PLLA and a high-molecular-weight PDLA blend.

## 3. Results and Discussions

**Figure 3:**
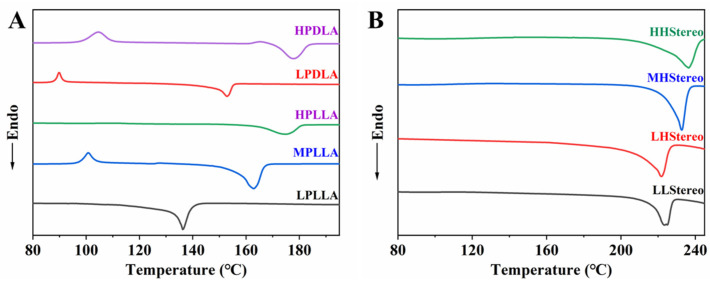
DSC curves of isomer PLA (**A**) and PLLA/PDLA blend films (**B**).

DSC was used to determine the melting temperature (T_m_) of all synthesized PLA. As shown in Figure 3A, the T_m_ of PLA increased monotonously with its molecular weight. The T_m_ of semicrystalline polymers can be enhanced up to a critical molecular weight, and afterward, the T_m_ is not affected noticeably. All homopolymers such as PLLA5, PLLA15, PLLA84, PDLA9, and PDLA90 depicted single endothermic peaks at around 136 °C, 163 °C, 175 °C, 153 °C, and 178 °C, respectively. The T_m_ of PLLA5 was much lower than the T_m_ of other PLA isomers. The DSC thermograms in Figure 3B have confirmed the formation of a stereocomplex of the blends between the PLLA and PDLA with different molecular weights. The detailed compositions of stereocomplexed and molecular weights of the PLLA and PDLA are summarized in Table 2.

Jun Shao et al. stated that when the molecular weight of PDLA was lower than that of PLLA, the crystallites were formed more because of the high mobility of PDLA, which results in a lower Tm for stereocomplexes even with high mobility [26]. Similarly, low molecular weight PDLA (10 k or less) exhibits higher mobility, and it could form a stereocomplex with each chain of PL**L**A. However, it requires a minimum chain length of each PLA isomer for forming stable stereocomplexes, and then LL and LH show similar T_sc_. As M_n_ of PLLA in the blend increases from 6 k (LH) to 15 k (MH), T_sc_ of MH is ca. 10 °C higher than that of LH, but T_sc_ of HH (M_n_ of PL**L**A: 85 k) slightly increases. Therefore, the critical M_n_ value of the isomer for stable complexes is around 15 k. However, the degree of complexation increases as M_n_ decreases, which is attributed to their enhanced mobility in solvents or melts. When the M_n_ of polymers, such as PLA, is higher, the polymer chains become longer and more entangled. As a result, the mobility of the polymer molecules decreases. This reduced mobility affects the ability of the polymer chains to form stereocomplexes, which are complexes formed between the PL**L**A and PDLA isomers. In the case of stereocomplex formation, the PL**L**A and PDLA isomers have a higher tendency to associate and form complexes at lower molecular weights because the chains have greater mobility. However, as the molecular weight increases to some critical point, the entanglement of the polymer chains restricts their movement and reduces their ability to form stereocomplexes.

However, stronger chain mobility and thin lamellar thickness of the low molecular weight PLA isomers (LLStereo) were observed to lower the T_sc_ for stereocomplexes. The lamellar thickness increased with molecular weight, and viscosity was also enhanced. The viscosity of the solution plays a prominent role in the formation of the stereocomplex [17,27,28]. The viscosity could fringe the mobility of polymer chains, and the formation of stereocomplexes became complicated. If the increase in viscosity of the polymer solution does not impede stereocomplex formation, it results in thicker lamellae and an increased Tm (melting temperature) of the stereocomplex, indicating the formation of only PLA stereocomplexes. However, as the molecular weight is further increased, the viscosity rises rapidly, which can limit mobility and hinder the interaction between the PLLA and PDLA molecular chains. Consequently, the formation of racemic crystallites becomes more challenging, and the Tm of the stereocomplex decreases. Figure 3A,B shows the thermal properties of different molecular weights of PLA isomers and PLLA/PDLA blend films. In this scenario, the PLLA and PDLA homochiral molecular chains tend to aggregate separately, forming PLA homocrystallites. As shown in Figure 3B, the highest T_sc_ (236.7 °C) was observed in the HHStereo blend, which can be attributed to the similarity in sequence length between both isomers within the same molecular weight range and the potential for the thickest stereocomplex crystal lamellae. The high molecular weight of the PLLA was estimated to be the high-molecular-weight chain of PDLA, which results in a prolonged crystal period and a higher T_sc_ of the stereocomplex. However, the T_m_ of LPLLA was much lower than other PLA isomers, but the T_sc_ of the blends has not been observed. It depicts that the chain length of LPLLA is long enough to complex with LPDLA and HPDLA.

**Figure 4:**
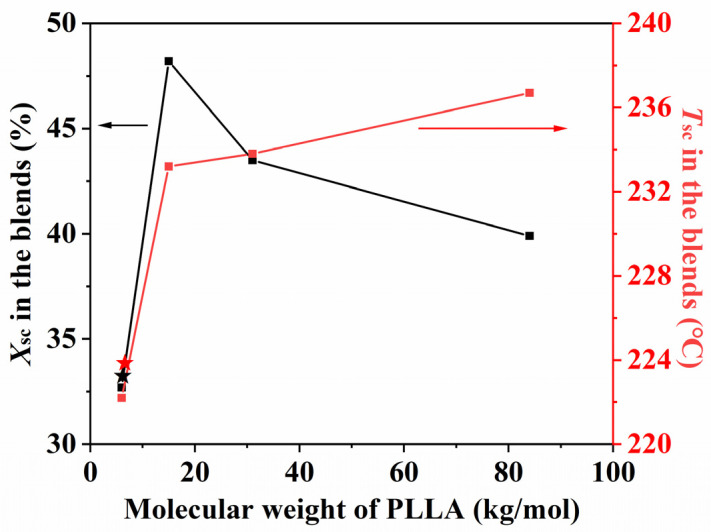
X_sc_ and T_sc_ of PLA stereocomplexes with different molecular weights, where M_n_ of PDLA was fixed to 90 k (X_sc_ and T_sc_ of LLStereo indicated by **★**).

In the PLLA/PDLA blend films (Figure 3), both T_sc_ are decreased at first and then rapidly increased when the molecular weight of PLLA and PDLA increased, while X_sc_ is first decreased, then increased, and again decreased as the molecular weight of PLLA and PDLA increased (Figure 4). T_sc_ was highest when the molecular weights of the PLLA and PDLA were 84 k and 90 k, respectively. When the stereocomplex with PLLA and PDLA has a low molecular weight, the nucleation and growth of the racemic crystallites appear to come to completion readily by solvent evaporation within a short time [29]. However, in the case of forming a stereocomplex (LHStereo) using PLLA and PDLA with different molecular weights, there is a possibility of diffusion from the low molecular weight compound to the high molecular weight compound. This diffusion can lead to the nucleation and growth of racemic crystallites. This observation supports the hypothesis that racemic crystallites are preferentially formed over homopolymer crystallites when the molecular weight of at least one compound in the blend of PLLA and PDLA is sufficiently small [30]. Alternatively, the blend of high-molecular-weight PLLA and PDLA may not be uniformly mixed at a microscopic level due to enthalpic repulsion between dissimilar high-molecular-weight molecules. However, if the viscosity does not hinder stereocomplex formation, the T_sc_ (stereocomplex melting temperature) increases as the molecular weight of the isomers increases, indicating successful stereocomplexation.

**Figure 5:**
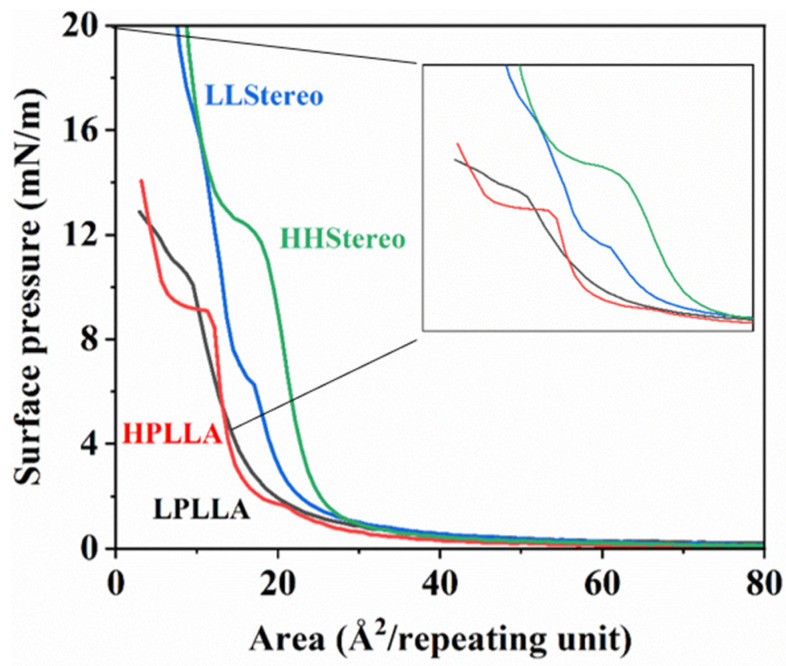
π-A isotherms of PLLA and stereocomplexed monolayers at pH 8.6.

The π-A isotherms, which depict the relationship between surface pressure and mean molecular area, provide insights into the lateral interactions between molecules in Langmuir monolayers. In Figure 5, the π-A isotherms for PLLA and stereocomplex monolayers on a sub-phase at pH 8.6 are presented. Consistent with previous studies [19,22,31,32,33], the π-A isotherm of PLLA monolayers exhibits a transition at approximately 10 mN/m. At surface pressures above 2 mN/m, the surface pressure of PLLA monolayers experiences a sharp increase, indicating a transition from a gaseous state to an expanded state of the film. However, in this study, the PLLA molecule (LPLLA) with a molecular weight of 10 k or less was not shown a specific transition region, and it exhibits a little expanded and compressible monolayer, occupied at 3 and 10 mN/m surface pressures at a limiting area of 8 and 16 Å^2^/repeating units. Additionally, both polymers illustrate more expanded and compressible monolayers, possessing at low surface pressure a limiting area of about 50 Å^2^/repeating units. The corresponding isotherm of high molecular weight (HPLLA) reveals a longer and flatter plateau than the PLLA with low molecular weight (LPLLA). Specifically, in the PLLA with high molecular weight (HPLLA), intense phase transition behavior was seen, in which the surface pressure was constant.

This difference was also evidenced in the stereocomplex curve. The corresponding isotherms of high-molecular-weight polymer blends (HHStereo) depict one-phase transition behavior above the PLLA homopolymers (12 mN/m). The phase transition intervenes at an approximately constant surface pressure with the longer and flatter plateau, which means that the area occupied by the polymer chains increased because of the interaction between the two molecules. Although the blend of low-molecular-weight isomer PLA (LL**S**tereo) shows an increment in the area occupied by the two oligomer molecules, the phase transition behavior was divided into two sections at 7 mN/m and 16 mN/m. More specifically, it shows a short plateau, and the transition is comparable to one of the second plateaus, signifying the surface pressure does not remain constant and the molecular chain is too short to form a complete stereocomplex (the limit of entanglement).

**Figure 6:**
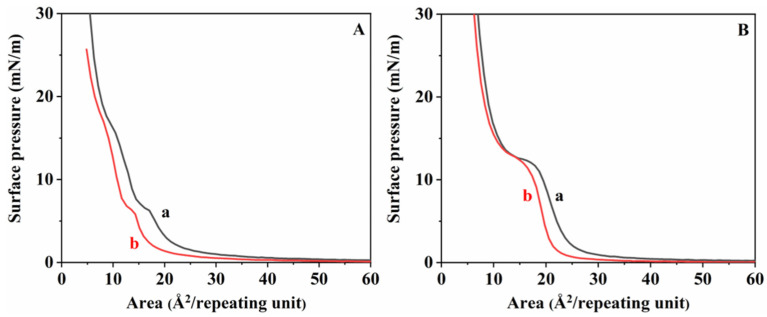
π-A isotherms of (**A**) LLStereo and (**B**) HHStereo at pH 8.6: a: before degradation; b: after enzymatic degradation.

Figure 6 depicts the comparison of the π-A curve of the existing stereocomplex by enzymatic degradation when the monolayer was subjected to two consequent compression expansions. As shown in Figure 6, a and b are the π-A curves of the stereocomplex before and after degradation, respectively. The first compression cycle was performed by compressing the monolayer film at a pressure of 4 mN/m. As expected, when degradation occurs in polymer chains, the original polymer chain breaks into smaller segments. In specific situations, these smaller segments dissolve into the water and catalyze hydrolysis, which decreases the occupied area. As shown in Figure 6A,B, the reduced area was significantly larger in LLStereo. Based on this, the stereocomplex between isomeric PLAs having a molecular weight of 10 k or less can retard the degradation rate more than in a homopolymer state but cannot escape the influence of molecular weight.

**Figure 7:**
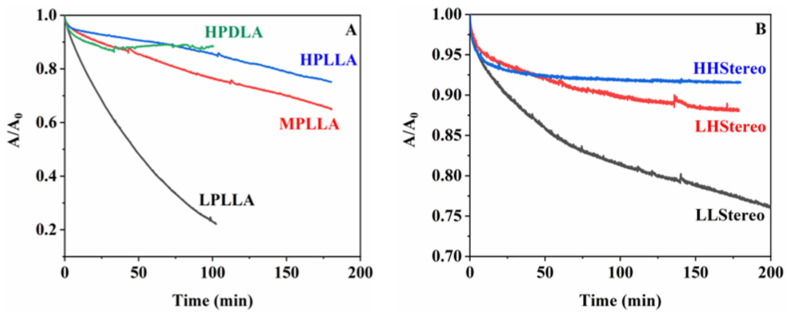
Area ratio vs. time of PLA homopolymer (**A**) and stereocomplex (**B**) at 4 mN/m on the subphase of pH 8.6 with proteinase K.

The degradation rate of polyester monolayers is primarily determined by two factors: the accessibility of subphase ions to the ester bonds and the strength of the bonds, leading to the formation of water-soluble oligomers [31,32,33]. Figure 7 illustrates that the hydrolyzed particles of low molecular weight disperse into the subphase, causing a decrease in the occupied area at a constant surface pressure. Therefore, the degradation rate is quantified as the ratio 1-A/A_0_ with respect to hydrolysis time, where A and A_0_ represent the occupied areas after degradation times, t, and at the start, respectively.

Enzymatic degradation of monolayers was conducted at a pH of 8.6. Figure 7 depicts the confirmation of the degradation behavior of the PLA and stereocomplex monolayers on the water subphase through the Langmuir system. Figure 7A,B illustrates the degradation behavior of homopolymer PLA and the PLA stereocomplexed monolayers, respectively. As we know, proteinase K can catalyze the degradation of l-lactyl chains in amorphous regions. These long l-lactyl-free chains in the amorphous regions can be enzymatically cleaved, although the folded chains in the crystalline regions are highly stable to enzymatic cleavage. Accordingly, the degradation occurred primarily in the amorphous regions of PLLA from proteinase K, but not in PDLA. The monolayers at high surface pressure are less dense than the amorphous state in bulk due to the very short chain folding structure at the air/water interface. The cleavage of the main chains in PLLA monolayers led to a decrease in molecular weight or chain length, and the resulting short oligomers are dissolved in water, which results in a rapid reduction in the area ratio, A/A_0_. However, it was confirmed that when the molecular weight was below 15 k, degradation occurred rapidly at the beginning stage, but the degradation rate gradually decreased significantly. As per previous studies, when degradation occurs at the initial stage on a surface of spin-coated polymer films on a slide glass or a silicon wafer, there is an uneven area, but as time passes, the polymer surface becomes smoother [34,35].

The majority of PLA degradation research was carried out using PLLA polymer, whereas, as per the author’s knowledge, only one report was reported using PDLA [36]. The formation of stereocomplexes within PLA isomers can largely retard the enzymatic degradation rate because of the presence of PDLA. PDLA is a polymer composed of D-lactic acid units and is not susceptible to enzymatic degradation by proteinase K. Enzymes that specifically target and degrade LLA sequences typically recognize the ester linkages present in the polymer chain. However, these enzymes are typically more effective at degrading PLLA than PDLA due to their higher susceptibility to hydrolysis. In particular, the presence of PDLA disrupted the adsorption or cleavage method of proteinase K. HPDLA monolayer shows changes in the A/A_0_ ratio (from 1 to 0.90) during an initial stage. It is due to the polymeric monolayers needing some time to be stable conformationally at the air/water interface when a surface pressure reaches a desired constant one. After that, monolayers are stable, and the A/A_0_ ratio is constant. However, LLStereo showed distinctly different characteristics as it contained very low-molecular-weight PLA isomers. As per the DSC results, LLStereo formed a complete single stereocomplex peak over 200 °C, but it showed a 25% reduction in the area occupied by the monolayer chain over 200 min, even though the decreasing rate of A/A_0_ is slower with time. Unlike the PLLA homopolymer, where complete degradation occurred in 100 min, LLStereo was able to delay the degradation rate if we consider that 50% of monolayers are LPLLA. The enzymatic degradation behavior of the LLStereo monolayers was surprised by the two-step kinetics as the area ratio, A/A_0_; it promptly decreased during an initial stage, which includes conformational stability and degradation of unstable complexed PLLA chains. However, the A/A_0_ behavior of HHStereo is very similar to that of HPDLA. The reason is that when the molecular weight exceeds a certain level, the stereocomplexed monolayers are stable for the enzyme due to a stereo effect.

**Figure 8:**
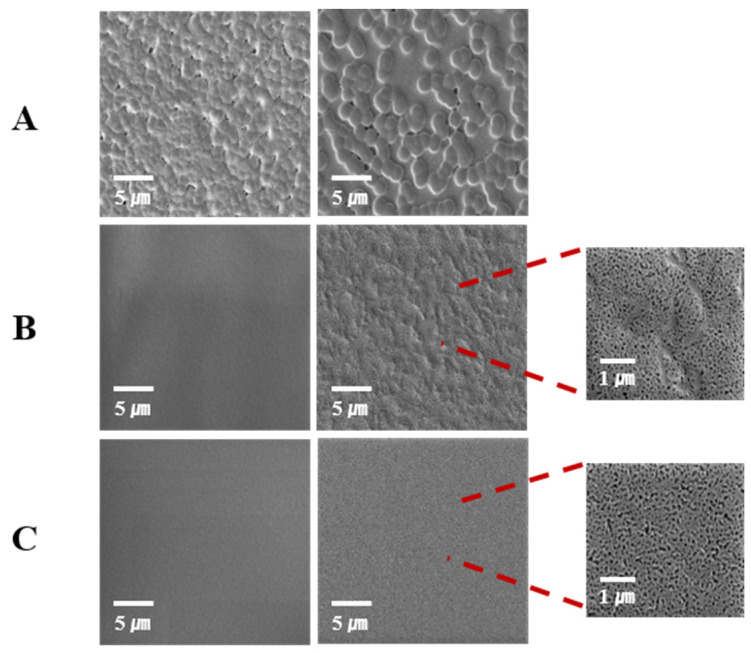
SEM images of PLLA/PDLA blends before (left) and after (right) enzymatic degradation (6 h); (**A**): LLStereo, (**B**): LHStereo, and (**C**): HHStereo.

Figure 8 depicts typical examples of SEM micrographs before and after the degradation of stereocomplexed samples with different molecular weights. It revealed that the LL sample had quite a different surface morphology, which included the spheres. These spheres might be evidence of rapid stereocomplex crystallite formation in solution or a polymer with very low M_n_ that is difficult to form clear films. Similar morphologies have been reported [37,38]. Low-molecular-weight polymers with highly concentrated solutions can form more spherulitic particles. The morphology displayed numerous voids, which could have arisen due to deficiencies in the stereocomplex microstructure or the shrinkage of microcrystallites during film drying. It is possible that the degradation of the amorphous phase between the stereocrystallites occurred at a faster rate compared to amorphous PLLA. This discrepancy may be attributed to the higher density of end-groups in the amorphous regions of crystalline stereocomplex PLA in contrast to amorphous PLLA [37,39,40,41,42]. LHStereo and HHStereo had a smoother surface before the degradation. However, many tiny holes were formed with degradation time as the partially uncomplexed PLLA chains were degraded since they were not included in stereocomplex crystallites.

## 4. Conclusions

In this study, PLA with different molecular weights was synthesized by the solution polymerization method to control molecular weight and chain architecture compared to conventional bulk polymerization. Results demonstrated that the molecular weights did not modulate the crystal structure of PLA and stereocomplexes, and the PLA stereocomplex was formed in all the blends. It has been confirmed through DSC analysis that stereocomplexation was thoroughly performed even at a molecular weight of 10 k or less. The melting temperatures in PLLA and PDLA increased uniformly as molecular weight increased. Although the T_sc_ in the PLLA/PDLA blends increased surprisingly, the highest T_sc_ was recorded at around 236 °C. The effect of the molecular weights of PLA on the enzymatic degradation behavior was studied at the molecular level with the Langmuir system. Homopolymer PLLA with a molecular weight of 10 k or less was degraded rapidly at the water/air interface, and the degradation rate was reduced significantly from 10 k or more. On account of confirming the degradation behavior by applying this to the stereocomplex, the degradation rate of a stereocomplex composed of a molecular weight of 10 k or less was delayed, but the degradation rate was still faster than that of the isomer PLA mixtures with a higher molecular weight. However, it was confirmed that when the molecular weight was greater than 15 k, degradation occurred rapidly at the beginning stage, but the degradation rate gradually decreased significantly. These results will expand the cognizance of PLA and PLA stereocomplex with different molecular weights, and superior thermal and degradation properties will contribute to more potential applications for PLA stereocomplex materials.

## Figures and Tables

**Table 1:** Synthesis of PLA with controlled molecular weight by varying the reaction parameters.

	Catalyst Amount (wt%)	Reaction Time (h)	Temperature (°C)	M_n_	M_w_	PDI	T_g_ (°C)	T_m_ (°C)
PLLA5 (LPLLA)	0.2	24	100	5.5 k	6.9 k	1.26	27.6	136.0
PLLA62	0.7	24	100	62 k	96 k	1.54		
PLLA84 (HPLLA)	1	24	100	84 k	140 k	1.66	61.6	175.2
PLLA83	3	24	100	83 k	142 k	1.7		
-	5	3	70	-	-	-		
PLLA15 (MPLLA)	5	12	100	15 k	19 k	1.28	53.4	163.3
PLLA28	5	24	100	28 k	46 k	1.63		
PDLA9 (LPDLA)	0.5	24	100	9 k	-	-	50.12	153.2
PDLA90 (HPDLA)	1	24	100	90 k	150 k	1.65	62.9	178.3
PDLA66	3	24	100	66 k	107 k	1.64		
PDLA74	5	24	100	74 k	126 k	1.7		
PDLA37	7	24	100	37 k	52 k	1.41		

Note: The first letters, L, M, and H, in the sample code indicate low, medium, and high molecular weights, respectively.

**Table 2:** Thermal properties of PLA blends measured by DSC.

Sample Code	T_m_ (°C)	Sources
LLStereo	223.8	LL-6K/LD-9K (50/50 by wt%)
LHStereo	222.2	LL-6K/HD-90K (50/50 by wt%)
MHStereo	233.2	ML-15K/HD-90K (50/50 by wt%)
HHStereo	236.7	HL-84K/HD-90K (50/50 by wt%)

## Data Availability

Not applicable.
